# Visual and Biochemical Evidence of Glycocalyx Disruption in Human Dengue Infection, and Association With Plasma Leakage Severity

**DOI:** 10.3389/fmed.2020.545813

**Published:** 2020-10-16

**Authors:** Phung Khanh Lam, Angela McBride, Duyen Huynh Thi Le, Trieu Trung Huynh, Hans Vink, Bridget Wills, Sophie Yacoub

**Affiliations:** ^1^Oxford University Clinical Research Unit, Ho Chi Minh City, Vietnam; ^2^Department of Global Health and Infection, Brighton and Sussex Medical School, Brighton, United Kingdom; ^3^Hospital for Tropical Diseases, Ho Chi Minh City, Vietnam; ^4^Department of Physiology, CardioVascular Research Institute Maastricht, Maastricht, Netherlands; ^5^Centre for Tropical Medicine and Global Health, University of Oxford, Oxford, United Kingdom

**Keywords:** dengue, glycocalyx, microvascular, perfused boundary region, syndecan, endocan

## Abstract

**Background:** Dengue is the most common arboviral infection globally; a minority of patients develop shock due to profound plasma leak through a disrupted endothelial barrier. Understanding of the pathophysiology underlying plasma leak is incomplete, but emerging evidence indicates a key role for degradation of the endothelial glycocalyx.

**Methods:** We conducted an observational study in Vietnam to evaluate the sublingual microcirculation using sidestream darkfield imaging in (1) outpatients with confirmed dengue (2) patients hospitalized with dengue and (3) outpatients with other febrile illness (OFI). We estimated the glycocalyx degradation by measuring the perfused boundary region (PBR hf) and an overall microvascular health score (MVHS) with the software application GlycoCheck^TM^ at enrolment, 48 h later and hospital discharge/defervescence. We measured plasma syndecan1 and endocan at the same time-points. We compared PBR hf, MVHS, syndecan1 and endocan, between (1) outpatients with confirmed dengue vs. OFI and (2) patients with dengue subdivided by clinical severity of plasma leak.

**Results:** We included 75 patients with dengue (41 outpatients, 15 inpatients, 19 in intensive care) and 12 outpatients with OFI. Images from 45 patients were analyzed using GlycoCheck^TM^. There was no significant difference in PBR hf or MVHS between outpatients with dengue and OFI. Median plasma syndecan1 was not significantly different in outpatients with dengue vs. OFI, while median plasma endocan was significantly lower among patients with dengue vs. OFI during the critical phase. In patients with dengue, PBR hf was higher in patients with Grade 2 vs. Grade 0 plasma leakage during the critical phase (PBR hf 1.96 vs. 1.36 μm for Grade 2 vs. Grade 0 plasma leakage on days 4–6, respectively, *p* < 0.001). Median levels of plasma syndecan1 and endocan were higher in Grade 2 vs. Grade 0 plasma leakage, especially during the critical phase (Syndecan1 2,613.8 vs. 125.9 ng/ml for Grade 2 vs. Grade 0 plasma leakage on days 4–6, respectively, *p* < 0.001, and endocan 3.21 vs. 0.16 ng/ml for Grade 2 vs. Grade 0 plasma leakage on days 4–6, respectively).

**Conclusions:** We present the first human *in vivo* evidence of glycocalyx disruption in dengue, with worse visual glycocalyx damage and higher plasma degradation products associated with more severe plasma leak.

## Introduction

Dengue virus (DENV) infection is the most common arboviral infection globally, with an estimated annual incidence of 96 million symptomatic infections (1). Although most infections are self-limiting, a minority progress to shock due to profound plasma leak through a disrupted endothelial barrier. The endothelial hyper-permeability usually terminates abruptly after ~48 h, with resorption of extravasated fluid into the intravascular space. Although understanding of the mechanisms underlying the onset and termination of plasma leak during dengue infection remains incomplete, the endothelial glycocalyx is emerging as central to the pathophysiology.

The endothelial glycocalyx is a negatively charged, ~0.5–5 μm, layer lining the microvasculature. It comprises a mesh of endothelial membrane-bound proteoglycans (such as syndecan-1 and glypicans), glycoproteins (cell surface receptors such as selectins and integrins), and glycosaminoglycans (GAGs - long, negatively-charged linear polysaccharides including heparan sulfate and chondroitin sulfate), with soluble plasma components (including albumin, and thrombomodulin) embedded within (2). The glycocalyx is a dynamic structure, whose components are constantly shed and replenished during normal blood flow in equilibrium with flowing macromolecules. It serves several key roles including maintenance of barrier function, prevention of leucocyte and platelet adhesion, modulation of clotting activation and mechano-transduction of shear stress to regulate endothelial nitric-oxide dependent vasodilation (2, 3).

Both *in vivo* and *in vitro* studies suggest that direct endothelial damage can occur through DENV NS1 Ag and host-immune mediated damage via TLR4 activation and vasoactive mediators, contributing to the disruption of endothelial integrity (4–6). NS1 antigen is secreted from DENV-infected cells into the circulation, from where it attaches to chondroitin sulfate and heparan sulfate (HS) (7). NS1 induces both cytokine-dependent and independent damage to the endothelial glycocalyx, including direct induction of endothelial heparanases (6). Previous clinical studies have reported high plasma and urine levels of HS in dengue virus infection (8, 9). However, it is not clear whether this elevation is due to specific cleavage of HS from its core proteoglycan syndecan-1 (SDC1), or whether there is more widespread glycocalyx damage with shedding of core proteoglycans.

Laboratory-based research on the pathophysiology underlying DENV induced glycocalyx disruption has predominantly been performed using endothelial cell monolayers, with visualization of the glycocalyx by transmission electron microscopy. However, the glycocalyx readily degrades without the biomechanical forces exerted by constant blood flow, and during dye/fixation processes in the laboratory; thus, the potential to translate these methods to study human microvascular pathophysiology during dengue infection is limited.

We previously investigated the sublingual microcirculation using videomicroscopy (side stream dark-field imaging, SDF) during acute dengue infection and demonstrated that perfusion and flow of the microcirculation is impaired proportionate to disease severity (10). Technological advances now permit assessment of glycocalyx damage by SDF. RBCs maintain a measurable distance from the microvessel endothelial cells, and this is thought to represent the endothelial glycocalyx layer; the diameter of the glycocalyx is measured by subtracting the diameter of the RBC column from the known internal diameter of the vessel from SDF videos. The GlycoCheck^TM^ system measures how deeply RBC can penetrate into the glycocalyx layer, which is reflected by the Perfused Boundary Region (PBR). A high value means deeper penetration and more damage to the glycocalyx. PBR hf is PBR estimated at fixed high flow level based on flow-PBR curves for diameter classes 5–25 microns, which is suggested to be a more accurate assessment of glycocalyx depth than measurement in capillaries without flow.

As glycocalyx damage has not been assessed in dengue patients before, we set out to re-analyze stored SDF images with the new Glycocheck technology. We compared glycocalyx degradation and biomarkers (SDC1 and endocan) between (1) patients with DENV infection and other febrile illnesses (OFI), and (2) patients with DENV infection with and without plasma leak. We hypothesized that the glycocalyx degradation would be (1) increased in patients with dengue vs. OFI and (2) increased in dengue patients with vs. without plasma leakage. We also hypothesized that PBR hf would correlate with plasma levels of SDC1 and endocan.

## Methods

We used a selection of stored SDF videos and plasma samples collected from patients recruited into an observational study on vascular function in dengue between 2013 and 2015; the full study protocol has been published elsewhere (10, 11). Briefly, this was a prospective observational study of adults and children >5 years presenting to two Vietnamese hospitals, the National Hospital for Tropical Diseases (NHTD), Hanoi and the Hospital for Tropical Diseases (HTD), Ho Chi Minh City. Two groups of patients were recruited, either (1) individuals presenting with a febrile illness consistent with dengue to outpatient health facilities within 72 h of fever onset, or (2) individuals hospitalized with dengue warning signs or severe dengue. At the HTD site, only patients admitted to Intensive Care Unit (ICU) were enrolled. Participants were followed daily until resolution of their acute illness. Hospital admission and individual case management were determined according to clinical need, with all interventions documented in the case report forms. Research blood samples were obtained and microvascular assessments including SDF imaging performed, at 4 time points: enrollment, 48 h later, hospital discharge/defervesence and follow-up between days 14–21.

Ethical approval was obtained from the Oxford Tropical Research Ethics Committee and the Ethics review Committee at NHTD and HTD. Written informed consent was obtained from all participants, or a parent/guardian of children.

## Laboratory Investigations

### Dengue Diagnostics

Reverse transcriptase PCR (RT-PCR) (12), an NS1 test (Platelia enzyme-linked immunosorbent assay; BioRad), and commercial IgM serology assays (Capture ELISA, Panbio) were used to confirm DENV infection. Patients were defined as having laboratory confirmed dengue if RT-PCR, NS1 antigen or DENV IgM assays were positive at enrolment, or if there was IgM seroconversion between paired serological samples at least 5 days apart. A diagnosis of OFI was assigned to outpatients with fever but no laboratory evidence of acute or recent dengue—i.e., if they were negative for dengue virus RT-PCR, NS1, and IgM on paired serology.

### Glycocalyx videomicroscopy Measurements

SDF videomicroscopy was performed by 3 investigators at the 4 time-points described above. Video clips were obtained at 3 different sublingual sites for 60 s, with attention to minimize pressure artifacts and reduce secretions. Images were acquired using a handheld videomicroscope with a x5 objective lens (MicroVision Medical, Amsterdam, Netherlands), which uses green light-emitting diode illumination at a wavelength (55 nm) that is absorbed by hemoglobin in the red blood cells (RBCs). The original video analysis was performed using AVA 3.2 software.

**Figure 1:**
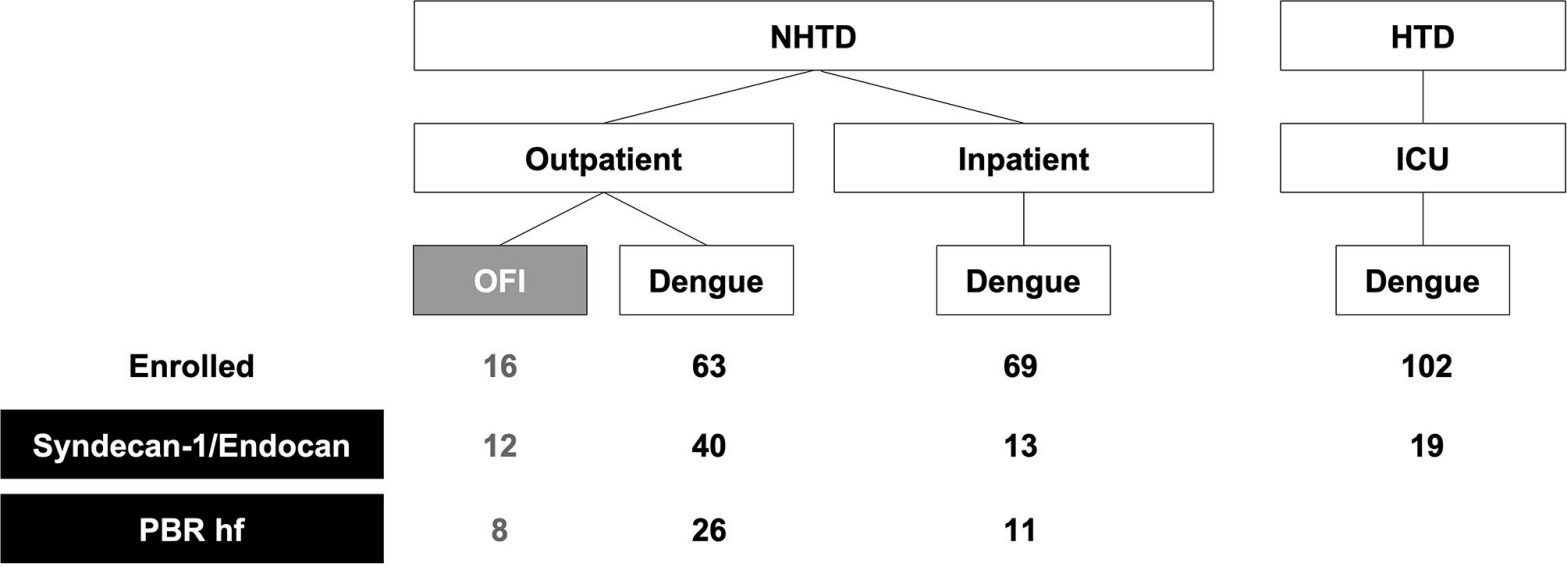
Study flowchart. A subset of patients prospectively enrolled into an observational study on vascular function in dengue between 2013 and 2015 had Syndecan-1, endocan, and PBR hf measured. Except for one outpatient with dengue and two inpatients with dengue, all other patients with PBR hf also had Syndecan-1 and endocan measured. NHTD, National Hospital for Tropical Diseases; HTD, Hospital for Tropical Diseases; ICU, Intensive Care Unit; OFI, Other Febrile Illnesses; PBR hf, Perfused Boundary Region high flow.

Due to a requirement for high quality images, only SDF videomicroscopy clips of sufficient quality were forwarded for GlycoCheck analysis (Figure 1); these video clips were analyzed by technicians blinded to diagnosis, disease severity and time-point at the Cardiovascular Research Institute of Maastricht University (CARIM), Netherlands. In addition to measurement of PBR hf, an overall microvascular assessment was made using the MicroVascular Health Score (MVHS). MVHS is derived using the PBR hf plus parameters representing the RBC filling (RBCF, red cell content of blood vessels), and valid vessel density (the subset of vessels that are functionally relevant, i.e., those with sufficient number of red cells for tissue perfusion). Lower values of MVHS reflect an overall worse microvascular health and higher PBR hf reflect a more degraded glycocalyx.

### Glycocalyx Biomarker Measurements

At the same 4 time-points, plasma levels of SDC1 and endocan were measured in patients whose images were forwarded for Glycocheck analysis, and additional patients from both NHTD and HTD sites with sufficient stored plasma (Figure 1). Measurements were made using specific sandwich ELISAs for SDC1 (Diaclone, Basancon, France) and endocan (Lunginnov, Lille, France) according to the manufacturers' instructions.

## Clinical End-Point Definitions

Different clinical outcomes of patients with DENV infection could be defined based on World Health Organization's guidelines (13–15). However, we focused on plasma leakage in this study. Patients with DENV infection were subdivided by plasma leakage grade as shown in Box 1. The haemoconcentration percentage [Δ Haematocrit (HCT)] was defined as (peak – baseline HCT/baseline HCT) x100. Clinical examination and chest X ray and/or ultrasound of the lungs and abdomen were performed to assess for extravascular fluid accumulation.

Box 1 **Plasma leakage grades in dengue virus infection.**

**Table:** 

**Grade 0**	**Grade 1**	**Grade 2**
No clinically significant plasma leak:	Moderate plasma leakage:	Severe plasma leakage:
Δ HCT <15%	ΔHCT 15–20%	ΔHCT > 20%
AND	AND/OR	AND/OR
**No** sign of fluid accumulation on clinical examination or x-ray/ultrasound	**Any** sign of fluid accumulation on clinical examination or x-ray/ultrasound	Shock or pleural effusion with respiratory compromise

## Statistical Analysis

First, the PBR hf, MVHS and glycocalyx biomarkers were compared between outpatients with confirmed dengue vs. OFI. Subsequently, the PBR hf, MVHS and glycocalyx biomarkers were compared between patients with dengue with varying degrees of plasma leakage using the mean difference between groups as the effect measure. As distributions of SDC1 and endocan are right-skewed, they were log-transformed prior to comparison. All analyses were based on linear regression models with the PBR hf and glycocalyx biomarkers as the outcome of interest, and dengue diagnosis or plasma leakage as covariates. For each covariate, an initial comparison with the outcome of interest used all measurements from the various time points assessed excluding the follow up values; separate analyses were subsequently performed for each disease phase: early (illness day 1–3); critical (illness day 4–6); recovery (illness day 7–13) and follow-up (after 13 days). Because most patients had multiple microcirculatory measurements performed, potentially with >1 measurement within each disease phase, robust sandwich errors based on working independence covariance structure derived from generalized estimating equations were used throughout the study. Comparisons were adjusted for age, sex, and illness day as appropriate. Of note, about half of the endocan values were below the limit of detection; therefore we conducted sensitivity analyses in which we imputed them using different values and investigated the impact on results. As results for endocan were consistent across scenarios in the sensitivity analysis, only the most conservative scenario (imputing below detection limit values with the detection limit of 0.156 ng/mL) is presented in the main results section. The full sensitivity analysis is reported in the Appendix.

Associations between PBR hf, MVHS and glycocalyx biomarkers in patients with dengue were assessed by means of partial correlations, controlling for the following potential confounding variables: age, sex, and illness day at measurement. The significance of partial correlations was assessed based on their Fisher transformation and corresponding bootstrap standard errors. The cluster bootstrap, which resamples patients rather than individual parameter values, accounted for multiple measurements per patient. To adjust for multiplicity informally, we used a significance level of 0.01 for all comparisons. All analyses were performed with the statistical software R (version 3.6.0) (16) and the companion package geepack (version 1.2.1) (17).

## Results

### Population

A total of 75 patients with dengue (41 recruited from outpatients, 15 inpatients, 19 in ICU) and 12 outpatients with OFI were included in this analysis (Table 1). High quality images from 45 of these patients (8 = with OFI and *n* = 37 with dengue with a range of plasma leak) were forwarded for the analysis of PBR hf. Figure 1 shows the number of patients included in each analysis.

**Table 1:** Characteristics at enrolment and clinical outcomes of patients included in this analysis.

	**OFI (*n* = 12)**		**Dengue (*n* = 75)**					
			**Outpatient (*n* = 41)**		**Inpatient (*n* = 15)**		**ICU (*n* = 19)**	
**Characteristics**	***n***	**Summary**	***n***	**Summary**	***n***	**Summary**	***n***	**Summary**
**At enrolment**								
Age (years)	12	24 (22, 28)	41	26 (22, 35)	15	26 (19, 36)	19	10 (7, 13)
Gender: female	12	6 (50)	41	20 (49)	15	9 (60)	19	9 (47)
Day of illness	12		41		15		19	
1–3		11 (92)		32 (78)		2 (13)		1 (5)
4–6		1 (8)		9 (22)		10 (67)		16 (84)
7–8		0 (0)		0 (0)		3 (20)		2 (11)
PCR positive	12	0 (0)	41	37 (90)	15	9 (60)	19	13 (68)
Platelet count (10^9^/L)	12	168 (147, 195)	39	144 (118, 176)	15	45 (29, 83)	18	26 (19, 34)
White blood cell count (10^9^/L)	12	10.3 (5.2, 12.5)	39	4.2 (3.0, 5.0)	15	4.6 (3.1, 6.2)	18	4.5 (2.8, 5.2)
Albumin (g/L)	12	44 (41, 46)	36	46 (42, 48)	6	32 (26, 36)	18	33 (28, 37)
Aspartate aminotransferase (IU/L)	12	16 (15, 29)	39	32 (26, 50)	9	128 (71, 163)	18	172 (126, 483)
Alanine aminotransferase (IU/L)	12	12 (10, 22)	40	24 (16, 43)	9	77 (34, 87)	18	102 (41, 342)
Creatinine (μmol/L)	12	93 (77, 107)	39	83 (73, 114)	10	72 (62, 89)	16	51 (46, 56)
**Outcome**								
WHO 2009 classification	–		41		15		19	
Dengue		–		26 (63)		1 (7)		0 (0)
Dengue with warning signs		–		12 (29)		8 (53)		3 (16)
Severe dengue		–		3 (7)		6 (40)		16 (84)
Plasma leakage grade		–	41		14		18	
Grade 0		–		33 (80)		6 (43)		1 (6)
Grade 1		–		6 (15)		2 (14)		1 (6)
Grade 2		–		2 (5)		6 (43)		16 (88)
Haemoconcentration >20%		–	41	2 (5)	12	3 (25)	16	11 (69)
Pleural effusion with respiratory compromise		–	41	0 (0)	15	3 (20)	19	5 (26)
Dengue shock syndrome		–	41	1 (2)	15	2 (13)	19	16 (84)

*Summary statistic is absolute count (%) for categorical variables and median (IQR) for continuous variables*.

*Haemoconcentration percentage was defined as (peak haematocrit—baseline haematocrit/baseline haematocrit) x100, “peak haematocrit” was the maximum of at least 3 haematocrit values in the acute illness (at least one of them was between days 4–7), and “baseline haematocrit” was the lowest value (in the following order) from the follow-up sample, a sample obtained within 72 h of fever onset, or (for hospitalized patients only) the discharge sample, provided no parenteral fluid was administered during the preceding 12 h*.

*OFI, Other Febrile Illnesses; ICU, Intensive Care Unit; PCR, Polymerase Chain Reaction*.

### Glycocalyx Videomicroscopy Assessment

**Figure 2:**
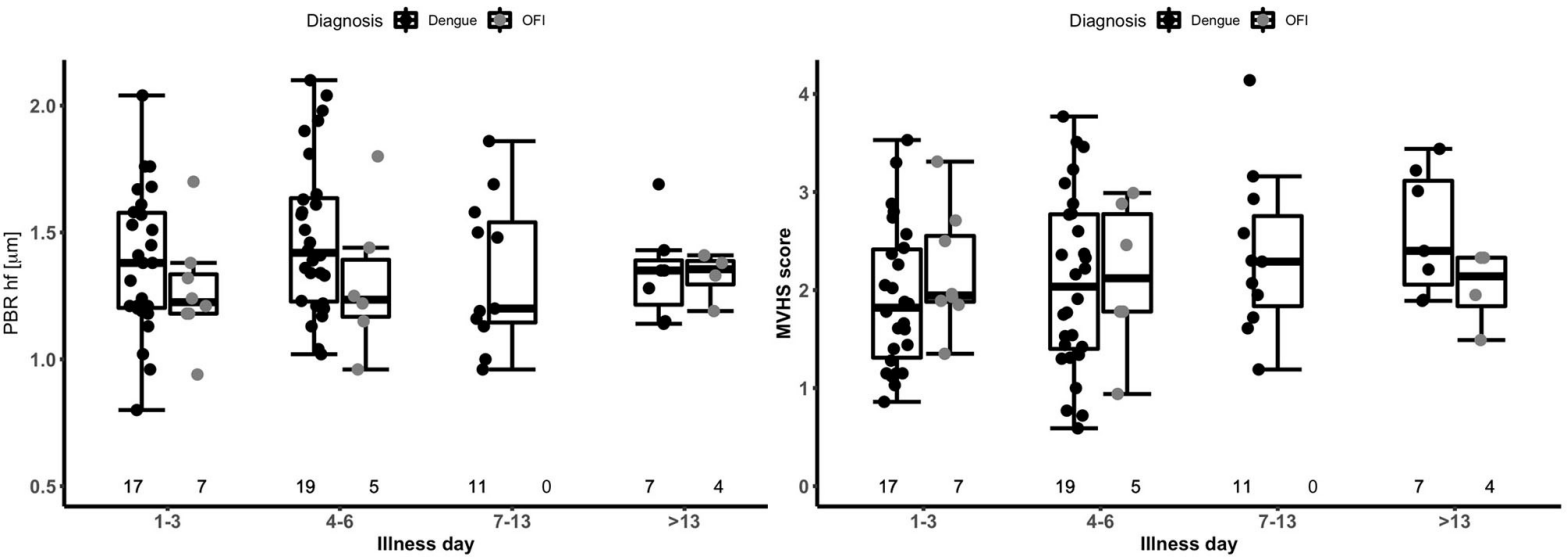
Boxplot of perfused boundary region and microvascular health score by disease phase in patients with dengue versus OFI. The short black line indicates the median value in each disease phase. The graph is based on 34 outpatients (26 dengue and 8 OFI) contributing 79 measurements. The numbers at the bottom of the graph indicate the number of patients in each group. PBR hf, Perfused Boundary Region high flow; MVHS, MicroVascular Health Score; OFI, Other Febrile Illness.

There were no statistically significant differences in PBR hf or MVHS (or of its components RBC filling, Valid vessel Density) between the dengue and OFI groups in pooled or illness day specific analyses (Table 2, lower panel, Figure 2). Collectively, these data indicate similar patterns of glycocalyx disturbance in the outpatient dengue and OFI patient groups. Of note, only two of the outpatients with dengue included in this analysis subsequently progressed to require hospitalization with Grade 2 plasma leakage.

**Table 2:** Glycocalyx parameters for outpatients with dengue and OFI by disease phase.

	**OFI (*n* = 12)**			**Dengue (*n* = 41)**					
**Time-point**	***n***	***N***	**Median (IQR)**	***n***	***N***	**Median (IQR)**	**MD**	**(95% CI)**	***p*-value**
**Glycocalyx videomicroscopy assessment**									
**PBR hf**									
**Overall**	**8**	**14**	**1.23 (1.18, 1.36)**	**26**	**65**	**1.39 (1.20, 1.61)**	**0.10**	**(−0.07, 0.28)**	**0.260**
Days 1–3	7	8	1.23 (1.18, 1.33)	17	26	1.38 (1.20, 1.58)	0.10	(−0.05, 0.26)	0.197
Days 4–6	5	6	1.23 (1.17, 1.39)	19	28	1.42 (1.23, 1.63)	0.13	(−0.13, 0.39)	0.320
Days 7–13	0	0	–	11	11	1.20 (1.14, 1.54)	–	–	–
Days >13	4	4	1.35 (1.29, 1.39)	7	7	1.35 (1.21, 1.39)	−0.02	(−0.10, 0.07)	0.708
**MVHS**									
**Overall**	**8**	**14**	**1.94 (1.80, 2.66)**	**26**	**65**	**1.95 (1.42, 2.60)**	**−0.10**	**(−0.62, 0.43)**	**0.714**
Days 1–3	7	8	1.94 (1.88, 2.55)	17	26	1.82 (1.31, 2.42)	−0.19	(−0.64, 0.27)	0.415
Days 4–6	5	6	2.12 (1.78, 2.78)	19	28	2.04 (1.40, 2.77)	0.00	(−0.75, 0.75)	0.999
Days 7–13	0	0	–	11	11	2.29 (1.83, 2.75)	–	–	–
Days >13	4	4	2.14 (1.83, 2.33)	7	7	2.40 (2.05, 3.12)	0.65	(0.17, 1.13)	0.008
**Glycocalyx biomarker measurements**									
**SDC1**									
**Overall**	**11**	**31**	**25.7 (8.1, 46.3)**	**40**	**113**	**80.9 (34.7, 206.9)**	**3.33**	**(0.95, 5.72)**	**0.006**
Days 1–3	10	14	26.4 (6.6, 53.7)	32	44	45.9 (33.2, 127.1)	3.52	(−0.22, 7.26)	0.065
Days 4–6	10	15	25.7 (8.6, 36.1)	34	54	119.2 (45.0, 208.6)	3.09	(0.33, 5.85)	0.028
Days 7–13	2	2	45.1 (33.6, 56.7)	14	15	1,021.8 (91.52, 1,434.0)	–	–	–
Days >13	6	6	12.7 (9.5, 27.9)	20	20	22.2 (14.2, 46.9)	−0.18	(−1.95, 1.59)	0.840
**Endocan**									
**Overall**	**12**	**34**	**0.97 (0.16, 2.44)**	**37**	**107**	**0.16 (0.16, 1.35)**	**−1.20**	**(−2.05,−0.35)**	**0.006**
Days 1–3	11	16	0.35 (0.16, 2.39)	29	40	0.16 (0.16, 0.83)	−0.79	(−1.95, 0.38)	0.186
Days 4–6	11	16	1.49 (0.21, 2.55)	31	50	0.16 (0.16, 0.61)	−1.55	(−2.57,−0.54)	0.003
Days 7–13	2	2	2.22 (1.65, 2.78)	16	17	2.30 (0.62, 3.23)	–	–	–
Days >13	7	7	0.19 (0.16, 0.40)	18	18	0.16 (0.16, 0.97)	0.18	(−0.84, 1.19)	0.733

*Since patients could contribute more than one measurement within and between groups, n, number of patients, and N, number of measurements in each group. Units are ng/ml for SDC1 and endocan, μm for PBR hf. The MDs represent mean differences in the corresponding biomarker between patients with dengue and OFI. For SDC1 and endocan, MDs represent mean differences in natural logarithm of SDC1 and endocan between patients with dengue and OFI*.

*“Overall” includes all values except for follow-up values, adjusted for age, sex and illness day. Other rows present comparisons for each disease phase, with adjustment for age and sex. All comparisons were based on linear regression model using generalized estimating equations with independence covariance structure to take into account multiple measurements per patient. Values that cannot be reliably estimated due to low number of measurements were indicated as –*.

*Values below the limit of detection for Endocan were imputed as 0.156 ng/mL (detection limit) in this analysis*.

*SDC1, Syndecan-1; CI, Confidence Interval; PBR hf, Perfused Boundary Region high flow; MVHS, MicroVascular Health Score; OFI, Other Febrile Illness; MD, Mean Difference; IQR, InterQuartile Range*.

**Figure 3:**
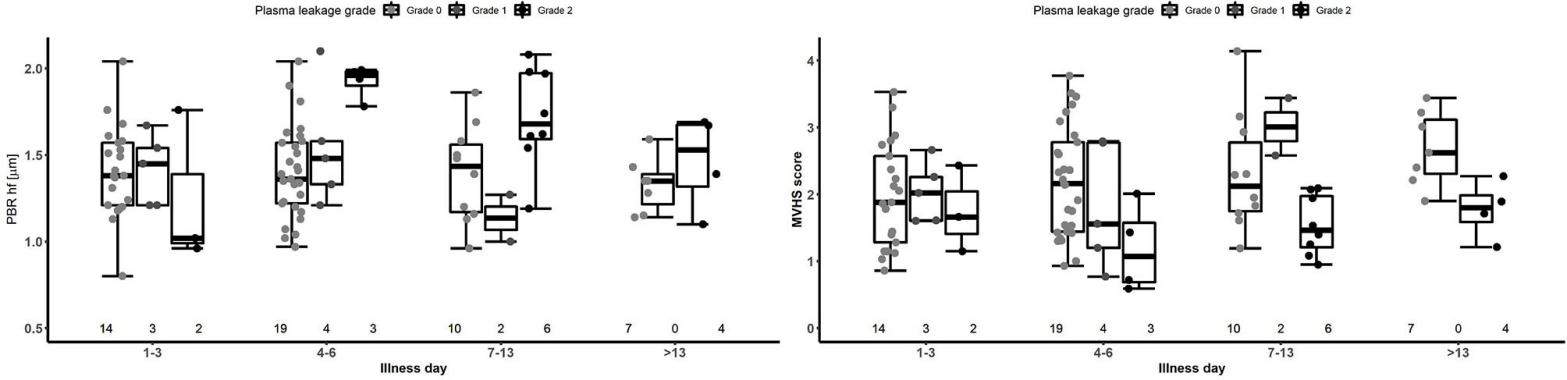
Boxplot of PBR hf and MVHS by plasma leakage grade and disease phase for patients with laboratory confirmed dengue. Dots represent individual values of PBR hf and MVHS in each group. The number represents the number of patients that contributed to each group. The graph is based on 36 patients contributing 87 measurements. PBR hf, Perfused Boundary Region high flow; MVHS, MicroVascular Health Score.

PBR hf values were obtained for a selection of 36 outpatients and inpatients with dengue at NHTD (Figure 3, Table 3, lower panel). Pooling all values up to illness day 13, the PBR hf value was higher in patients with Grade 2 vs. Grade 0 plasma leakage, which was driven by a marked difference in PBR hf during the critical phase [PBR hf 1.96 vs. 1.36 μm for Grade 2 vs. Grade 0 plasma leakage on days 4–6, respectively, mean difference 0.43 μm (95%CI 0.25–0.61) *p* < 0.001]. There were no statistically significant differences between patients with other plasma leakage grades in other illness day categories. There were insufficient measurements taken at follow-up to permit meaningful comparison by plasma leakage grade. For MVHS, pooled analysis suggests a significantly lower score in patients with Grade 2 compared to patients with Grade 0 plasma leak [mean difference of −0.53 (95%CI −0.82, −0.25), *p* < 0.001], as well as reductions in its other components, including RBCF [75.1 vs. 68.8%, mean difference of −2.82 (95% CI −5.6 to 0.03, *p* = 0.047), and valid density (273 vs. 173 sites/mm^2^, mean difference of −62.0 (95%CI −106.6, −17.5), *p* = 0.006]. However, there was no clear indication of a difference by illness day group.

**Table 3:** Glycocalyx parameters for outpatient and inpatients with laboratory confirmed dengue by plasma leakage grade and disease phase.

			**Leakage grade 0**			**Leakage grade 1**			**Leakage grade 2**			**Leakage grade 1 vs. 0**			**Leakage grade 2 vs. 0**
**Time-point**	***N***	***N***	**Median (IQR)**	***n***	***N***	**Median (IQR)**	***n***	***N***	**Median (IQR)**	**MD**	**(95% CI)**	***p*-value**	**MD**	**(95% CI)**	***p*-value**
**Glycocalyx videomicroscopy assessment**															
**PBR hf**															
**Overall**	**24**	**60**	**1.38 (1.20, 1.57)**	**5**	**12**	**1.39 (1.21, 1.55)**	**7**	**15**	**1.76 (1.58, 1.98)**	**0.04**	**(−0.08, 0.16)**	**0.502**	**0.20**	**(0.04, 0.36)**	**0.013**
Days 1–3	14	21	1.38 (1.21, 1.57)	3	5	1.45 (1.21, 1.54)	2	3	1.02 (0.99, 1.39)	0.04	(−0.14, 0.23)	0.640	−0.12	(−0.48, 0.23)	0.501
Days 4–6	19	29	1.36 (1.22, 1.57)	4	5	1.48 (1.33, 1.58)	3	4	1.96 (1.90, 1.98)	0.10	(−0.15, 0.35)	0.424	0.43	(0.25, 0.61)	<0.001
Days 7–13	10	10	1.44 (1.17, 1.56)	2	2	1.14 (1.07, 1.20)	6	8	1.68 (1.59, 1.97)	−0.15	(−0.39, 0.08)	0.205	0.24	(−0.02, 0.51)	0.069
Days >13	7	7	1.35 (1.21, 1.39)	0	0	–	4	4	1.53 (1.32, 1.67)	–	–	–	–	–	–
**MVHS**															
**Overall**	**24**	**60**	**2.08 (1.44, 2.75)**	**5**	**12**	**2.14 (1.59, 2.69)**	**7**	**15**	**1.40 (1.11, 1.97)**	**0.08**	**(−0.26, 0.42)**	**0.630**	**−0.55**	**(−0.85**, **−0.26)**	**<0.001**
Days 1–3	14	21	1.88 (1.28, 2.57)	3	5	2.02 (1.61, 2.26)	2	3	1.66 (1.40, 2.04)	0.03	(−0.47, 0.54)	0.892	−0.30	(−1.40, 0.80)	0.589
Days 4–6	19	29	2.16 (1.44, 2.78)	4	5	1.56 (1.20, 2.77)	3	4	1.07 (0.69, 1.57)	−0.20	(−1.06, 0.66)	0.656	−0.84	(−1.62, −0.06)	0.034
Days 7–13	10	10	2.12 (1.75, 2.77)	2	2	3.01 (2.79, 3.23)	6	8	1.32 (1.19, 1.97)	0.68	(−0.45, 1.81)	0.236	−0.61	(−1.11, −0.10)	0.018
Days >13	7	7	2.62 (2.30, 3.12)	0	0	–	4	4	1.80 (1.58, 1.98)	–	–	–	–	–	–
**Glycocalyx biomarker measurements**															
**SDC1**															
**Overall**	**38**	**111**	**124.8 (41.6, 281.1)**	**8**	**25**	**212.9 (26.4, 450.3)**	**24**	**73**	**1,220.1 (268.5, 3,149.9)**	**−0.29**	**(−2.49, 1.91)**	**0.796**	**2.67**	**(1.16, 4.17)**	**<0.001**
Days 1–3	25	33	46.9 (36.4, 134.0)	6	10	30.6 (23.2, 43.8)	3	4	117.0 (66.5, 484.2)	−0.85	(−1.80, 0.11)	0.082	0.87	(−1.79, 3.53)	0.523
Days 4–6	34	56	125.9 (47.7, 220.3)	6	9	228.6 (212.9, 450.3)	20	34	2,613.8 (1,182.7, 3,910.9)	−0.71	(−5.22, 3.80)	0.757	3.76	(1.61, 5.91)	<0.001
Days 7–13	14	22	678.2 (139.4, 1,136.9)	5	6	990.81 (422.69, 1,583.94)	23	35	668.5 (181.3, 1,803.3)	1.20	(−1.11, 3.51)	0.310	1.67	(−0.13, 3.47)	0.070
Days >13	19	19	20.5 (13.2, 49.8)	3	3	63.30 (41.10, 140.44)	20	20	34.43 (12.63, 90.10)	0.98	(−0.50, 2.46)	0.193	3.69	(0.37, 7.00)	0.029
**Endocan**															
**Overall**	**34**	**100**	**0.16 (0.16, 2.27)**	**7**	**22**	**0.58 (0.16, 6.28)**	**16**	**47**	**4.00 (0.85, 7.75)**	**1.09**	**(−0.03, 2.20)**	**0.056**	**1.68**	**(0.69, 2.66)**	**<0.001**
Days 1–3	22	29	0.16 (0.16, 1.39)	6	10	0.16 (0.16, 0.16)	3	4	0.48 (0.16, 1.60)	−0.72	(−1.82, 0.38)	0.200	1.30	(−1.20, 3.80)	0.307
Days 4–6	30	51	0.16 (0.16, 1.07)	5	7	3.91 (1.37, 6.85)	13	21	3.21 (0.82, 7.84)	2.40	(1.07, 3.72)	<0.001	2.14	(0.73, 3.55)	0.003
Days 7–13	15	20	2.38 (0.70, 6.58)	4	5	7.12 (2.85, 12.60)	15	22	5.38 (2.55, 8.17)	1.94	(0.03, 3.86)	0.047	1.69	(0.48, 2.90)	0.006
Days >13	17	17	0.20 (0.16, 1.14)	2	2	0.16 (0.16, 0.16)	13	13	0.91 (0.62, 1.55)	−1.47	(−2.29, −0.65)	<0.001	0.80	(−0.48, 2.07)	0.222

*Since patients could contribute more than one measurement within and between groups, n, number of patients, and N, number of measurements in each group. Units are ng/ml for SDC1 and endocan, μm for PBR hf,. The mean difference (MD) represents difference in plasma level/score between patients with plasma leakage grades (grade 1 compared to grade 0 and grade 2 compared to grade 0). For SDC1 and endocan, MDs represent mean differences in natural logarithm of SDC1 and endocan between patient groups*.

*“Overall” includes all values except for follow-up values, adjusted for age, sex and day of illness. Other rows present comparisons for each disease phase, with adjustment for age and sex. All comparisons were based on linear regression using generalized estimating equations with independence covariance structure to take into account multiple measurements per patient. Values cannot be reliably estimated due to low number of measurements were indicated as –*.

*Values below the limit of detection for Endocan were imputed as 0.156 ng/mL (detection limit) in this analysis*.

*For SDC1 and endocan, the analyses were based on 70 dengue patients (including 18 ICU patients); whereas for PBR hf and MVHS, the analyses were based on 36 dengue patients (not including any ICU patients)*.

*SDC1, Syndecan-1; PBR hf, Perfused Boundary Region high flow; MVHS, MicroVascular Health Score; IQR, InterQuartile Range; MD, Mean Difference; CI, Confidence Interval*.

**Figure 4:**
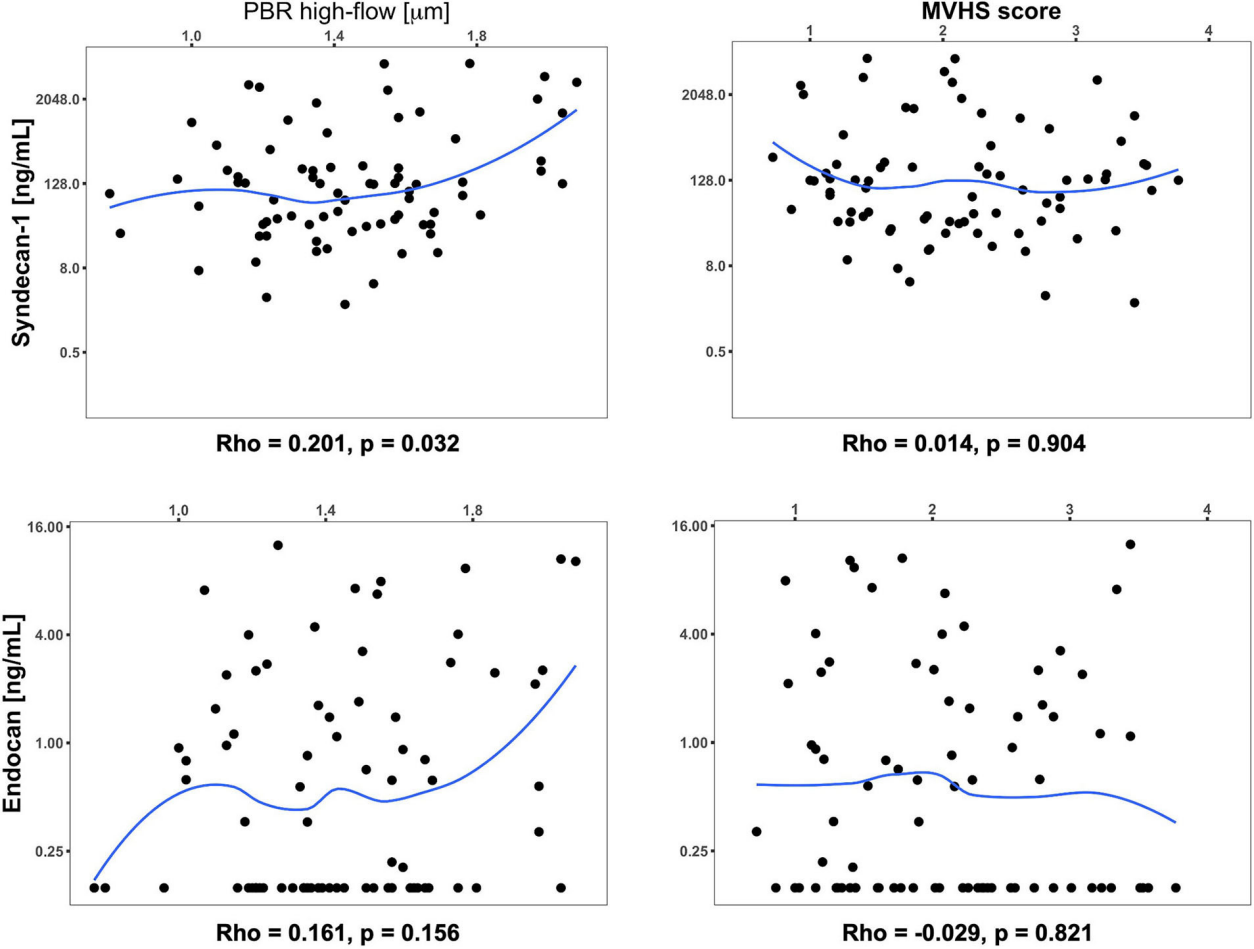
Scatterplot and partial correlation coefficient (Rho) between the respective two parameters of interest controlling for age, sex, and day of illness at measurement. The significance of partial correlations (p) was assessed based on their Fisher transformation and corresponding bootstrap standard errors. Values below the limit of detection for Endocan were imputed as 0.156 ng/mL (detection limit) in this analysis.

We did not find significant partial correlations (using *P* < 0.01) between the levels of core proteoglycans SDC1 and endocan, and the microcirculatory parameters PBR hf and MVHS, after controlling for age, sex and illness phase (Figure 4). The correlation of PBR hf and SDC1 approached significance with *p* = 0.032.

### Glycocalyx Biomarker Measurements

Median plasma SDC1 was higher among outpatients with dengue compared to OFI at all time-points, but after adjustment for multiple comparisons, the differences did not reach statistical significance (Table 2, upper panel). In contrast, median plasma endocan was significantly lower among patients with dengue compared to OFI during the critical phase (illness day 4–6).

**Figure 5:**
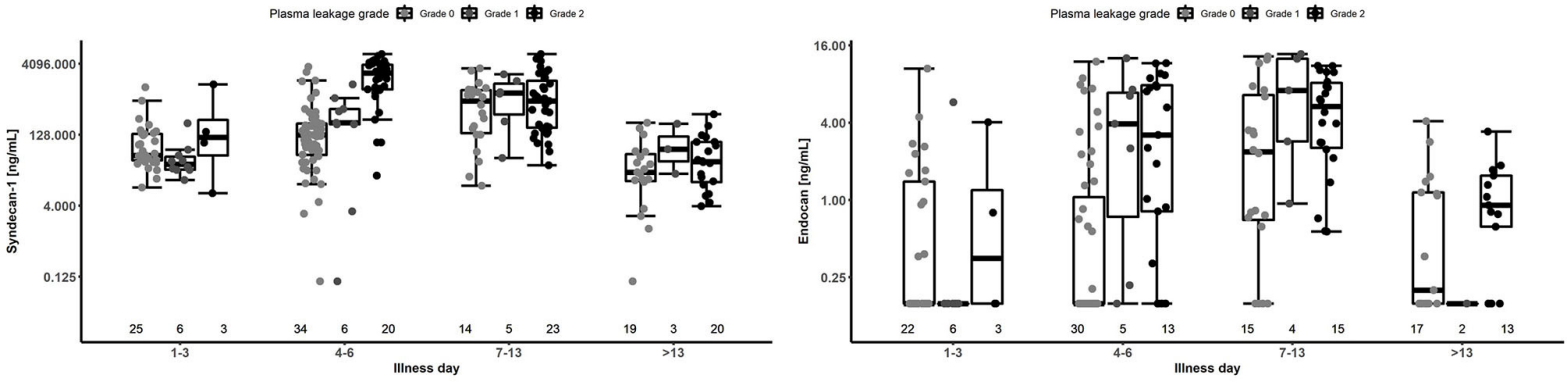
Boxplot of plasma SDC1 and endocan measurements by illness day and plasma leakage grade for patients with laboratory confirmed dengue. Dots represent individual values of SDC1 and endocan in each group. The number below represents the number of patients that contributed to each group. The SDC1 graph is based on 70 patients contributing 209 measurements, and the endocan graph is based on 57 patients contributing 169 measurements. Values below the limit of detection for Endocan were imputed as 0.156 ng/mL (detection limit) in this analysis.

Results for patients with laboratory confirmed dengue categorized by plasma leakage severity are presented in Table 3, upper panel. Median plasma SDC1 levels were higher in patients with Grade 2 plasma leakage compared to Grade 0 plasma leakage at all time-points, the difference being most marked during the critical phase (SDC1 2,613.8 vs. 125.9 ng/ml for Grade 2 vs. Grade 0 plasma leakage on day 4–6, *p* < 0.001). Similarly, median plasma endocan levels were higher in patients with Grade 2 plasma leakage compared to Grade 0 plasma leakage at all time-points, especially during the critical phase (Endocan 3.21 vs. 0.16 ng/ml for Grade 2 vs. Grade 0 plasma leakage on day 4–6, *p* = 0.003) and recovery phase (Endocan 5.38 vs. 2.38 ng/ml for Grade 2 vs. Grade 0 plasma leakage on day 7–13, *p* = 0.006) (Figure 5, Table 3).

## Discussion

In this observational study, we have shown that the glycocalyx is significantly degraded in patients with the most severe clinical manifestations of plasma leak due to dengue infection. In addition, plasma levels of two glycocalyx core proteoglycans, SDC1 and endocan, were elevated in association with clinical severity of plasma leak. However, there were not significant differences when comparing glycocalyx breakdown between outpatients with dengue, most of whom had mild plasma leak, and other non-severe febrile illnesses.

Suwarto et al. (18) also found an association between plasma SDC1 levels and severity of plasma leak in adults with dengue in Indonesia. However, median SDC1 level was higher among patients with severe plasma leakage during the critical phase in our study (median SDC1 2,613.8 ng/ml, IQR 1,182–3,910 ng/ml in our study, vs. median 468.87 ng/ml, IQR 278.7–538.06 ng/ml reported by Suwarto et al.), despite use of the same ELISA in both. This difference might be explained by relatively earlier plasma sampling by Suwarto et al., or by more severe disease among our study participants.

SDC1 shedding has been demonstrated in other forms of shock; patients admitted with haemorrhagic shock due to major trauma had significantly raised SDC1 levels compared to healthy controls (median 545 vs. 37 ng/ml) (19). Several studies have also found SDC1 levels to be raised in proportion with sepsis severity, as recently summarized by Uchimido et al. (20). Although our control population did not include patients with other severe febrile illnesses, the levels of SDC1 in patients with severe plasma leakage in our study were several fold higher than in most other published studies of shock (20); this may provide a plausible explanation for why patients with dengue shock appear to experience a more marked plasma leakage than those with haemorrhagic and septic shock.

In this study, we found that plasma endocan levels were higher during the critical phase in dengue with severe plasma leakage vs. no plasma leakage. However, endocan levels reported in the published literature for patients with septic shock appear to be higher when compared to patients in our study with dengue shock. Mihajlovic et al. reported endocan levels in patients with septic shock [mean: 6.11 ± 7.8 ng/ml, *n* = 12 (21)], compared to a median of 4.0 ng/ml in patients with dengue shock in this study. Indeed, when comparing outpatients with dengue vs. OFI over all time-points, we found that median endocan levels were higher in patients with OFI compared to dengue. This suggests that although endocan is elevated in proportion to plasma leak severity, it may be a poorly specific biomarker for dengue-induced glycocalyx breakdown.

A possible reason for targeted SDC1 shedding over endocan could be related to dengue viral binding to heparan sulfate (HS), SDC1's GAG side chain. We have previously shown that patients with dengue have higher levels of plasma and urinary HS (8). The results from the current study suggest that DENV does not cause isolated HS release into the circulation after viral binding, but that more widespread damage to the glycocalyx occurs with core proteoglycan release into the circulation in addition to the GAG side chains (22). Viral or NS1 binding to HS may result in enzymatic degradation of HS, with subsequent increase in the susceptibility of SDC1 to proteolytic cleavage by matrix metalloproteinases (MMP's) (23). An alternative explanation may be leakage of shed endocan through the disrupted endothelial barrier, leading to an artifactual lowering of plasma levels compared to OFI in mild disease.

Taken together, our results suggest there is targeted glycocalyx damage in dengue with severe plasma leakage, with damage to core proteoglycans in addition to GAG sidechains, and a simultaneous decrease in the depth of the glycocalyx. Preservation of this layer by preventing shedding of glycocalyx components, or accelerating repair may be a potential therapeutic target (20). Although no therapeutic agents to abrogate glycocalyx breakdown have yet been investigated in humans with dengue, interest is growing in the potential to translate therapeutics developed for other indications; these include strategies to inhibit heparanase activity [modified heparins, Sulodexide (24)], downregulate MMP activity to reduce proteoglycan shedding [S1P analogs (25, 26)], use intravenous fluids (IV) which may accelerate glycocalyx repair (27) [albumin (28), fresh frozen plasma (29)], or avoid bolus administration of IV fluids to prevent further damage to a fragile glycocalyx (30). However, screening of such therapeutic strategies for vascular leak in dengue requires an improvement *in vitro* models to evaluate glycocalyx integrity.

The extent to which the observed reduction in glycocalyx depth in the sublingual microcirculation mirrors changes in the glycocalyx in other microvascular beds is uncertain. However, unlike other flaviviruses, dengue appears to cause systemic, rather than organ-specific microvascular damage (31), thus in dengue the sublingual changes may be an adequate sentinel for glycocalyx degradation in other microvascular beds. In this study, glycocalyx thickness was most diminished in patients with severe plasma leak during the critical phase, when plasma leak is already clinically apparent. Therefore, the SDF technique might not be useful for early risk prediction, but it may serve as an additional surrogate to other clinical and biological endpoints to evaluate the impact of novel therapeutic strategies on glycocalyx integrity during future research (32). Utilizing software updates for the glycocheck analysis allowing automated real-time evaluation of the glycocalyx and future development of a point-of-care assays for syndecan would provide clinically useful bedside tools for assessment of microvascular damage and plasma leakage in dengue patients.

A strength of this study is that the microcirculation was evaluated early during the disease course, which allowed the evolution and timing of the glycocalyx pathology to be studied. In addition, studying the changes *in vivo* eliminated the problems mentioned above with respect to *in vitro* models of glycocalyx integrity. A limitation of this study is that our control population did not include patients with other severe infections such as septic shock; it is possible that the increase in PBR hf we observed in patients with severe plasma leak is also non-specific. Direct comparison of PBR hf with simultaneous, serial plasma measurement of several glycocalyx components would help to clarify whether the pathology is common to patients with other forms of shock, or specific to dengue; the answers may aid translation of therapeutic candidates.

In conclusion, we have shown visual and biochemical evidence of glycocalyx degradation in patients with dengue virus infection. Further, the most marked disruption was found in patients with severe clinical manifestations of plasma leakage. Exploring therapeutic strategies to prevent further damage to, or accelerate restoration of the glycocalyx layer may help to reduce morbidity associated with plasma leak in dengue; SDC1 and/or PBR hf may serve as useful surrogate endpoints in future clinical trials.

## Data Availability Statement

The raw data supporting the conclusions of this article will be made available by the authors, without undue reservation.

## Ethics Statement

The studies involving human participants were reviewed and approved by Oxford Tropical Research Ethics Committee and the Ethics review Committee at NHTD and HTD. Written informed consent to participate in this study was provided by the participants' legal guardian/next of kin.

## Author Contributions

SY and BW designed the study. SY carried out the Video microscopy. AM and PL analyzed the results and wrote the manuscript. TH coordinated the study. DL carried out the laboratory studies. HV coordinated the glycocheck analysis. All authors contributed to the article and approved the submitted version.

## Conflict of Interest

HV is the Chief Scientific advisor of GlycoCheck BV in The Netherlands. SY receives consultancy fees from Janssen pharmaceuticals for dengue antiviral development and as a member of the ROCHE Advisory Board on Severe Dengue. The remaining authors declare that the research was conducted in the absence of any commercial or financial relationships that could be construed as a potential conflict of interest.
