# Supplementary material for: Visual and Biochemical Evidence of Glycocalyx Disruption in Human Dengue Infection, and Association With Plasma Leakage Severity
Source: Front Med (Lausanne). 2020 Oct 16;7:545813. doi: 10.3389/fmed.2020.545813 (PMC7596352; doi:10.3389/fmed.2020.545813)
Supplement: Supplementary file 1 [file Table_1.docx]

Appendix

**Sensitivity analysis for Endocan**

There was a considerable number of Endocan level values below detection limit (BDV) (detection limit is 0.156 ng/mL): 88/166 (53%) of values in the comparison between dengue and OFI, and 86/205 (42%) of values in the comparison between plasma grades. We investigated impact of BDV on results in 3 different situations: imputing them with the highest value (detection limit), imputing them with the moderate value (half of detection limit), and imputing them with the very low value (0.001, we did not use 0 as we need natural logarithm of endocan values). In all 3 situations, our sensitivity analyses (presented in Table S1 and S2) suggest a lower level of endocan in dengue patients compared to OFI group and higher level of endocan in dengue patients with higher leakage grade, both with largest difference during the critical period (day 4-6). Of note, most of BDV were in the dengue group (75/88, 85%) in the comparison between OFI and dengue, and most of BDV were in the grade 0 group (62/83, 75%) in the comparison between leakage grades. As results are consistent in all 3 situations, we only reported the most conservative scenario (BDV were imputed with detection limit, option 1) in the main text.

**Table S1.** *Sensitivity analysis for Endocan in outpatients with dengue and OFI by illness phase. In this analysis, there were 88 Endocan values below detection limit (0.156 ng/mL) [13 in the OFI group and 75 in the dengue group].*

*Since patients could contribute more than one measurement within and between groups, n=number of patients, and N=number of measurements in each group. Unit of endocan level is ng/mL. The mean difference (MD) represents mean difference in natural logarithm of endocan between patients with dengue and OFI.*

*“Overall” includes all values except for follow-up values, adjusted for age, sex and illness day. Other rows present comparisons for each illness day category, with adjustment for age and sex. All comparisons were based on linear regression model using generalized estimating equations with independence covariance structure to take into account multiple measurements per patient. Values cannot be reliably estimated due to low number of measurements were indicated as -.*

*Option 1: below detection limit values (BDV) were imputed as 0.156 ng/mL (detection limit); Option 2: BDV were imputed as 0.078 ng/mL (half of detection limit); Option 3: BDV were imputed as 0.001.*

***Abbreviations:*** *Confidence Interval; OFI, Other Febrile Illness; MD, Mean Difference; IQR, InterQuartile Range.*

|  |  |  | **OFI (n=12)** |  |  |  | **Dengue (n=41)** | **MD** |  |  |
| --- | --- | --- | --- | --- | --- | --- | --- | --- | --- | --- |
| **Time-point** | **n** | **N** | **Median (IQR)** |  | **n** | **N** | **Median (IQR)** | **(Dengue vs OFI)** | **(95% CI)** | **p value** |
| **Option 1** |  |  |  |  |  |  |  |  |  |  |
| **Overall** | **12** | **34** | **0.97 (0.16, 2.44)** |  | **37** | **107** | **0.16 (0.16, 1.35)** | **-1.20** | **(-2.05, -0.35)** | **0.006** |
| Days 1-3 | 11 | 16 | 0.35 (0.16, 2.39) |  | 29 | 40 | 0.16 (0.16, 0.83) | -0.79 | (-1.95, 0.38) | 0.186 |
| Days 4-6 | 11 | 16 | 1.49 (0.21, 2.55) |  | 31 | 50 | 0.16 (0.16, 0.61) | -1.55 | (-2.57, -0.54) | 0.003 |
| Days 7-13 | 2 | 2 | 2.22 (1.65, 2.78) |  | 16 | 17 | 2.30 (0.62, 3.23) | - | - | - |
| Days >13 | 7 | 7 | 0.19 (0.16, 0.40) |  | 18 | 18 | 0.16 (0.16, 0.97) | 0.18 | (-0.84, 1.19) | 0.733 |
| **Option 2** |  |  |  |  |  |  |  |  |  |  |
| **Overall** | **12** | **34** | **0.97 (0.08, 2.44)** |  | **37** | **107** | **0.08 (0.08, 1.35)** | **-1.56** | **(-2.55, -0.57)** | **0.002** |
| Days 1-3 | 11 | 16 | 0.35 (0.08, 2.39) |  | 29 | 40 | 0.08 (0.08, 0.83) | -1.08 | (-2.47, 0.31) | 0.128 |
| Days 4-6 | 11 | 16 | 1.49 (0.19, 2.55) |  | 31 | 50 | 0.08 (0.08, 0.61) | -1.98 | (-3.20, -0.75) | 0.002 |
| Days 7-13 | 2 | 2 | 2.22 (1.65, 2.78) |  | 16 | 17 | 2.30 (0.62, 3.23) | - | - | - |
| Days >13 | 7 | 7 | 0.19 (0.08, 0.40) |  | 18 | 18 | 0.08 (0.08, 0.97) | 0.05 | (-1.27, 1.38) | 0.937 |
| **Option 3** |  |  |  |  |  |  |  |  |  |  |
| **Overall** | **12** | **34** | **0.97 (0.00, 2.44)** |  | **37** | **107** | **0.00 (0.00, 1.35)** | **-3.82** | **(-5.75, -1.89)** | **<0.001** |
| Days 1-3 | 11 | 16 | 0.35 (0.00, 2.39) |  | 29 | 40 | 0.00 (0.00, 0.83) | -2.92 | (-5.79, -0.05) | 0.046 |
| Days 4-6 | 11 | 16 | 1.49 (0.17, 2.55) |  | 31 | 50 | 0.00 (0.00, 0.61) | -4.66 | (-7.33, -1.99) | <0.001 |
| Days 7-13 | 2 | 2 | 2.22 (1.65, 2.78) |  | 16 | 17 | 2.30 (0.62, 3.23) | - | - | - |
| Days >13 | 7 | 7 | 0.19 (0.00, 0.40) |  | 18 | 18 | 0.00 (0.00, 0.97) | -0.71 | (-4.37, 2.94) | 0.702 |

**Table S2.** *Sensitivity analysis for Endocan in patients with laboratory confirmed dengue, by plasma leakage grade and illness day. The analyses were based on 70 dengue patients (including 18 ICU patients). In this analysis, there were 83 Endocan values below detection limit (0.156 ng/mL) [62 in the grade 0 group, 12 in the grade 1 group, and 9 in the grade 2 group].*

*Since patients could contribute more than one measurement within and between groups, n=number of patients, and N=number of measurements in each group. Unit of endocan level is ng/mL. The mean difference (MD) represents difference in natural logarithm of plasma level of endocan between patients with plasma leakage grades (grade 1 compared to grade 0 and grade 2 compared to grade 0).*

*“Overall” includes all values except for follow up values, adjusted for age, sex and day of illness. Other rows present comparisons for each DOI category, with adjustment for age and sex. All comparisons were based on linear regression using generalized estimating equations with independence covariance structure to take into account multiple measurements per patient. Values cannot be reliably estimated due to low number of measurements were indicated as -.*

*Option 1: below detection limit values (BDV) were imputed as 0.156 ng/mL (detection limit); Option 2: BDV were imputed as 0.078 ng/mL (half of detection limit); Option 3: BDV were imputed as 0.001.*

***Abbreviations:*** *IQR, InterQuartile Range; MD, Mean Difference; CI, Confidence Interval.*

|  |  |  | **Leakage grade 0** |  |  | **Leakage grade 1** |  |  | **Leakage grade 2** | **Leakage grade 1 vs. 0** | | | **Leakage grade 2 vs. 0** | | |
| --- | --- | --- | --- | --- | --- | --- | --- | --- | --- | --- | --- | --- | --- | --- | --- |
| **Time-point** | **n** | **N** | **Median (IQR)** | **n** | **N** | **Median (IQR)** | **n** | **N** | **Median (IQR)** | **MD** | **(95% CI)** | **p value** | **MD** | **(95% CI)** | **p value** |
| **Option 1** |  |  |  |  |  |  |  |  |  |  |  |  |  |  |  |
| **Overall** | **34** | **100** | **0.16 (0.16, 2.27)** | **7** | **22** | **0.58 (0.16, 6.28)** | **16** | **47** | **4.00 (0.85, 7.75)** | **1.09** | **(-0.03, 2.20)** | **0.056** | **1.68** | **(0.69, 2.66)** | **<0.001** |
| Days 1-3 | 22 | 29 | 0.16 (0.16, 1.39) | 6 | 10 | 0.16 (0.16, 0.16) | 3 | 4 | 0.48 (0.16, 1.60) | -0.72 | (-1.82, 0.38) | 0.200 | 1.30 | (-1.20, 3.80) | 0.307 |
| Days 4-6 | 30 | 51 | 0.16 (0.16, 1.07) | 5 | 7 | 3.91 (1.37, 6.85) | 13 | 21 | 3.21 (0.82, 7.84) | 2.40 | (1.07, 3.72) | <0.001 | 2.14 | (0.73, 3.55) | 0.003 |
| Days 7-13 | 15 | 20 | 2.38 (0.70, 6.58) | 4 | 5 | 7.12 (2.85, 12.60) | 15 | 22 | 5.38 (2.55, 8.17) | 1.94 | (0.03, 3.86) | 0.047 | 1.69 | (0.48, 2.90) | 0.006 |
| Days >13 | 17 | 17 | 0.20 (0.16, 1.14) | 2 | 2 | 0.16 (0.16, 0.16) | 13 | 13 | 0.91 (0.62, 1.55) | -1.47 | (-2.29,-0.65) | <0.001 | 0.80 | (-0.48, 2.07) | 0.222 |
| **Option 2** |  |  |  |  |  |  |  |  |  |  |  |  |  |  |  |
| **Overall** | **34** | **100** | **0.08 (0.08, 2.27)** | **7** | **22** | **0.58 (0.08, 6.28)** | **16** | **47** | **4.00 (0.85, 7.75)** | **1.22** | **(-0.08, 2.53)** | **0.067** | **1.97** | **(0.81, 3.13)** | **<0.001** |
| Days 1-3 | 22 | 29 | 0.08 (0.08, 1.39) | 6 | 10 | 0.08 (0.08, 0.16) | 3 | 4 | 0.44 (0.08, 1.60) | -0.99 | (-2.33, 0.35) | 0.146 | 1.63 | (-1.59, 4.84) | 0.322 |
| Days 4-6 | 30 | 51 | 0.08 (0.08, 1.07) | 5 | 7 | 3.91 (1.37, 6.85) | 13 | 21 | 3.21 (0.82, 7.84) | 2.89 | (1.28, 4.49) | <0.001 | 2.54 | (0.87, 4.20) | 0.003 |
| Days 7-13 | 15 | 20 | 2.38 (0.70, 6.58) | 4 | 5 | 7.12 (2.85, 12.60) | 15 | 22 | 5.38 (2.55, 8.17) | 2.18 | (0.15, 4.20) | 0.035 | 1.92 | (0.59, 3.26) | 0.005 |
| Days >13 | 17 | 17 | 0.20 (0.08, 1.14) | 2 | 2 | 0.08 (0.08, 0.08) | 13 | 13 | 0.91 (0.62, 1.55) | -2.01 | (-3.08,-0.95) | <0.001 | 1.05 | (-0.56, 2.66) | 0.203 |
| **Option 3** |  |  |  |  |  |  |  |  |  |  |  |  |  |  |  |
| **Overall** | **34** | **100** | **0.00 (0.00, 2.27)** | **7** | **22** | **0.58 (0.00, 6.28)** | **16** | **47** | **4.00 (0.85, 7.75)** | **2.09** | **(-0.48, 4.66)** | **0.111** | **3.82** | **(1.44, 6.20)** | **<0.001** |
| Days 1-3 | 22 | 29 | 0.00 (0.00, 1.39) | 6 | 10 | 0.00 (0.00, 0.00) | 3 | 4 | 0.40 (0.00, 1.60) | -2.69 | (-5.56, 0.17) | 0.066 | 3.65 | (-4.08, 11.38) | 0.355 |
| Days 4-6 | 30 | 51 | 0.00 (0.00, 1.07) | 5 | 7 | 3.91 (1.37, 6.85) | 13 | 21 | 3.21 (0.82, 7.84) | 5.97 | (2.52, 9.41) | <0.001 | 5.03 | (1.59, 8.48) | 0.004 |
| Days 7-13 | 15 | 20 | 2.38 (0.70, 6.58) | 4 | 5 | 7.12 (2.85, 12.60) | 15 | 22 | 5.38 (2.55, 8.17) | 3.64 | (0.66, 6.61) | 0.017 | 3.40 | (1.03, 5.77) | 0.005 |
| Days >13 | 17 | 17 | 0.20 (0.00, 1.14) | 2 | 2 | 0.00 (0.00, 0.00) | 13 | 13 | 0.91 (0.62, 1.55) | -5.41 | (-8.10,-2.72) | <0.001 | 2.62 | (-1.35, 6.59) | 0.196 |
